# Development of liquid chip for functional genes in foxtail millet (*Setaria italica*) and association analysis of related traits

**DOI:** 10.3389/fpls.2026.1784690

**Published:** 2026-03-24

**Authors:** Xi Zhang, Yuchuan Zhang, Xiaoling Zhang, Meng Wang, Mingrui Zhao, Ziyang Lv, Xianrui Wang, Yuhao Yuan, Baili Feng

**Affiliations:** 1 State Key Laboratory of Crop Stress Biology for Arid Areas, College of Agriculture, Northwest A & F University, Shaanxi, China; 2 Chifeng Academy of Agricultural Sciences, Chifeng, Inner Mongolia, China; 3 State Key Laboratory of Wheat and Maize Crop Science, College of Agronomy, Henan Agricultural University, Henan, China

**Keywords:** biotechnology, foxtail millet, genotyping chip, precision breeding, SNP

## Abstract

Foxtail millet (*Setaria italica*) is a nutrient-rich, drought-tolerant cereal crop with significant potential for promoting sustainable agriculture. However, the lack of specialized, cost-effective genotyping tools has limited the development of breeding technology. We developed a liquid-phase functional single nucleotide polymorphism (SNP) chip utilizing Genotyping by Target Sequencing (GBTS) technology. This customized chip integrates 59 functionally verified SNPs from 30 key genes governing critical agronomic traits, including selenium accumulation, plant architecture, yield, stress tolerance, and grain quality. We validated the approach on a panel of 192 foxtail millet accessions. The chip demonstrated a high average call rate of 98.53% and revealed robust genotype-phenotype associations for traits such as fertility, leaf color, leaf sheath color, and seed color. Furthermore, population structure analysis clearly delineated modern cultivars from landraces and admixed groups, highlighting its utility in genetic diversity studies. This robust, scalable, and cost-effective genotyping tool is poised to significantly accelerate marker-assisted selection and precision breeding programs in foxtail millet, with broader implications for genetic research in other Poaceae species.

## 1 Introduction

Foxtail millet (*Setaria italica*), with a domestication history in China dating back approximately 10,500 years, ranks among the most ancient cultivated cereals (X. Yang et al., 2012). Due to its drought tolerance, salt-alkali tolerance, low-fertility soil tolerance, and nutritional richness, this crop holds significant importance for food security in arid and semi-arid regions (Bettinger et al., 2010; Sharma and Niranjan, 2018). Compared to other major cereal crops such as wheat (*Triticum aestivum*) and rice (*Oryza sativa*), foxtail millet is considered highly amenable to domestication. This is attributed to its small diploid genome (~515Mb; 2n=2x=18) which is the same as that of rice, short generation time, inbreeding nature (Doust et al., 2009; Lata et al., 2013), and the extensive validation of the reliability of the reference genome (Setaria italica v2.2) in multiple published studies (Bennetzen et al., 2012; Zhang et al., 2012). Due to the presence of the C_4_-specific spatial carbon concentration mechanism and close phylogenetic relationship with important crops such as maize and sorghum, it is regarded as an ideal model cereal for studies on photosynthetic metabolism, physiological mechanisms, genome evolution, and molecular genetics of stress tolerance (Wang et al., 2025). Genetic studies in foxtail millet can provide theoretical insights for improving stress resistance and high-yield breeding in Poaceae crops.

The foxtail millet germplasm conserved within China’s National Genebank, while extensive and diverse, poses a major bottleneck to breeding advancement due to its overwhelming reliance on traditional landraces and a corresponding deficit of improved modern varieties. Bridging this gap requires a paradigm shift in breeding methodology, focusing on innovations that streamline the breeding cycle, lower costs, and expedite the delivery of high-performing cultivars to farmers.

The liquid-phase functional gene chip is a novel genotyping technology based on GBTS, offering high detection throughput, low cost, and flexible design. Different from traditional microarray platforms constrained by predefined polymorphisms, Genotyping by Target Sequencing (GBTS) leverages second-generation sequencing technology (Campbell et al., 2015). This approach employs designed primers or probes to capture specific genomic regions, which are subsequently subjected to PCR amplification and deep sequencing (Samorodnitsky et al., 2015).This approach is also particularly suitable for targeted detection of functional genes or trait-associated loci because it relies on probe-target hybridization. The platform can simultaneously interrogate up to 45,000 SNP loci in a single run. By utilizing Genotyping by Target Sequencing (GBTS), this liquid-chip platform demonstrates notable versatility, compatibility with diverse sequencing instruments, operational efficiency and streamlined data processing. These advantages have led to its widespread adoption in genetic research, including population genetics, genetic map construction, genome-wide association studies (GWAS), QTL mapping, and gene discovery (Xiang et al., 2023; Yang et al., 2023). To date, the methodology has been successfully implemented in over 100 species, with prominent applications in soybean (Yang et al., 2023), wheat (Unterseer et al., 2014; Xiang et al., 2023), maize (Guo et al., 2021) and pepper (Li et al., 2024). The continuing limitations of current whole-genome sequencing platforms highlight a clear need for technological advancement. While targeted sequencing and solution capture have seen notable improvements, optimizing overall systems is crucial for achieving reliable DNA variant identification, cost-effective workflows, and scalable implementation in genomics research and molecular breeding (Burridge et al., 2018; Johnson et al., 2019).

To advance precision breeding in foxtail millet, we constructed a targeted genotyping panel using the GenoBaits liquid-phase SNP platform (Verlouw et al., 2021). Our marker selection strategy prioritized loci with functionally characterized alleles. The resulting panel encompasses 59 validated SNPs spanning four key trait categories: selenium content, plant architecture, yield components, and stress tolerance. The chip was meticulously designed through a stringent selection process focusing on loci with firmly established phenotypic effects from map-based cloning or well-characterized breeding materials. The selection of candidates for the SNP array was based on a genome-wide polymorphism survey. This survey utilized resequencing data from a globally sourced panel of 192 foxtail millet accessions, which provided a foundation for the chip design (Li et al., 2022; Luo et al., 2023; Xing et al., 2016). Extensive validation across diverse germplasm accessions demonstrated the chip’s exceptional performance, with high SNP call rates and reliable genotyping efficiency. This innovative genotyping platform provides researchers and breeders with a powerful, cost-efficient tool for advancing marker-assisted selection and precision breeding in foxtail millet, with particular promise for enhancing the crop’s value through improved selenium content.

## 2 Materials and methods

### 2.1 Plant source

The 192 foxtail millet germplasm resources were obtained from the Chifeng Academy of Agricultural Sciences (Chifeng, Inner Mongolia, China). These varieties are deposited in the China National Germplasm Resources Center and are distributed across different regions of China, mainly in the northwest. The accessions from China were specifically sourced from Inner Mongolia, Heilongjiang, Jilin, Liaoning, and Shanxi, including 88 wild accessions and 104 cultivated varieties. These accessions cover major ecological types of foxtail millet in China, including arid and semi-arid region ecotypes, ensuring the representativeness of genetic diversity. **Supplementary Materials** provides detailed information on these 192 millet varieties.

### 2.2 Material preparation

**Figure 1:**
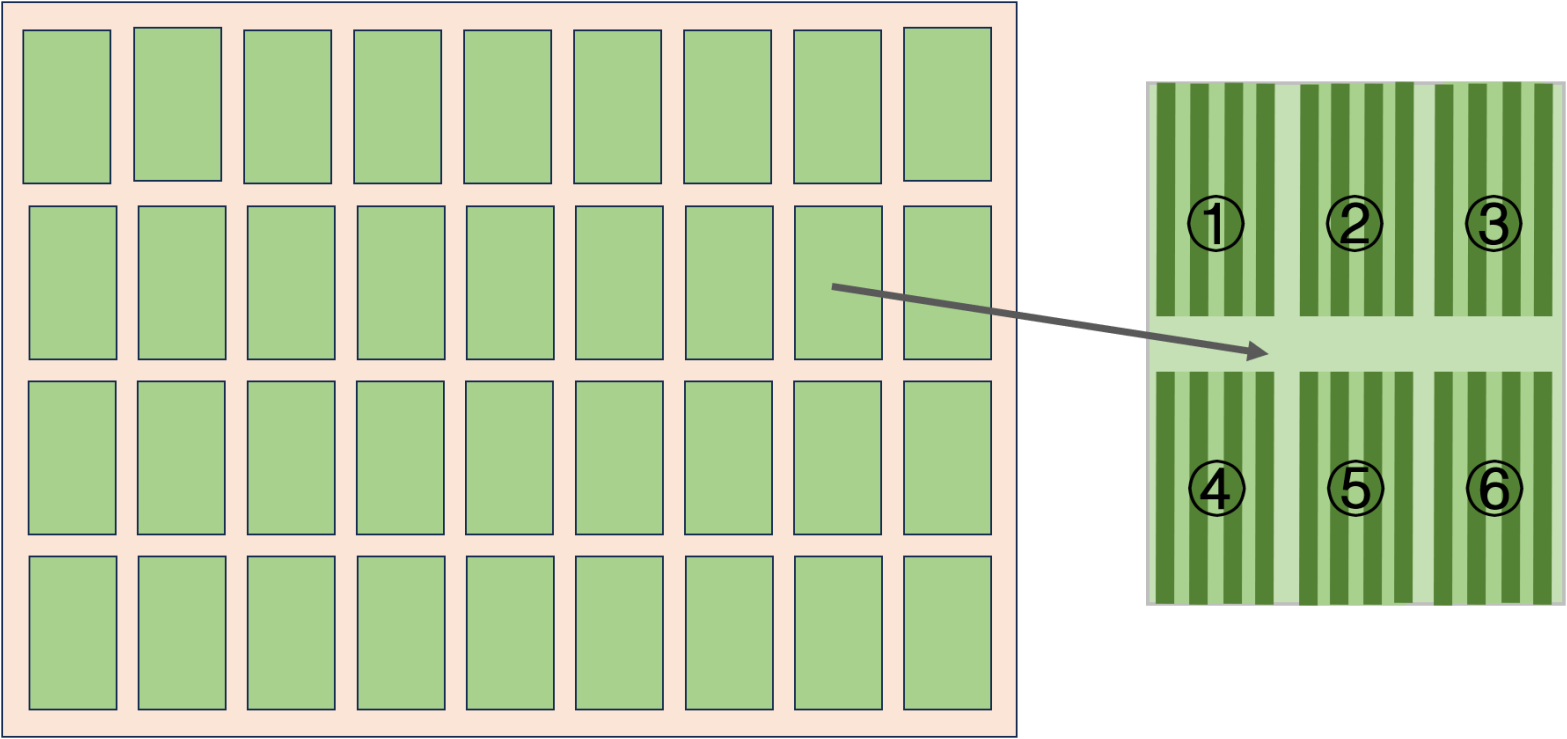
192 diagrams illustrating the cultivation of millet germplasm resources. The yellow part in the left picture is the non-cultivated area, and the green part is the cultivated area. In the right picture, the dark part is the planting row, with four rows for each variety, and the light part is the aisle.

The field experiment was located in the experimental site of Northwest A & F University (109.7 E, 38.3 N, 1,080 m altitude) in Yulin, Shaanxi Province, China, from April to October, during two consecutive years (2024 and 2025). This area belongs to the hilly and gully region of the Loess Plateau, with an average annual rainfall of 400 mm and a typical arid and semi-arid continental monsoon climate. Soil moisture content was adjusted to 20% through irrigation prior to planting. The field experiment used a single-factor completely randomized design. A total of 46 plots were used to accommodate 192 foxtail millet varieties. All genotypes were randomly distributed among plots by means of a random number table and each variety was arranged in four rows, each 2 meters in length (**Figure 1**). Conventional field management was applied throughout the growing period. The same material was placed in different plots over two years to reduce the environmental variation caused by the location of the communities. For all the measured phenotypic traits, we randomly selected 10 representative plants in each plot for determination. For each trait, the value used for association analysis was first calculated as the mean of 10 individuals per plot, and then averaged across the two years to obtain a single value per accession. Therefore, the final data points of each trait represent the comprehensive performance of the germplasm on the basis of two growing seasons and multiple individuals.

### 2.3 Field phenotypic trait determination

Phenotypic evaluation was conducted in vegetative period and harvest period, mainly concentrated in May and September. During the growth period, the following traits were evaluated: heading date, leaf attitude (LF), sheath color, and floral organ development. At maturity, plant height (PH), panicle weight (PW), panicle development, panicle compactness, seta length, and thousand-grain weight (KGW) were measured. After harvest, grain length, grain width, grain diameter, grain area, grain perimeter, grain roundness, grain length-to-width ratio, and thousand-grain weight (KGW) were measured using an automatic seed testing instrument (SC-G, Wan Shen Testing Co., China). Color parameters (L, a, b*) were measured with a colorimeter (WSF, Shanghai Precision Science Instrument Co., Ltd.).

These 14 agronomic traits were directly assessed in the field. The heading date was recorded when 50% of plants in experimental plots had fully emerged panicles. The total growth period was defined as the number of days from emergence to when 90% of grains reached mature coloration. Plant height was measured from the base of the main stem to the base of the first panicle branch at maturity. Leaf attitude was classified based on the angle between the blade and stem: Erect (<30°), Semi-Erect (30°-60°), Horizontal (60°-90°), or Drooping (>90°). Qualitative traits were visually evaluated using the maximum similarity principle. Panicle type was recorded based on the predominant shape in each plot and categorized as Spindle, Cylindrical, or Club-shaped. Panicle compactness was rated as Loose, Medium, or Tight based on tactile evaluation of mature panicles. Sheath color was assessed between the first and fifth leaf stages. Subsequently, at the 4–5 leaf stage, we selected leaves that were free from biological and abiotic stress and recorded the color of their upper surfaces. Both indicators were classified as yellow-green, green, or purple. Seta length was estimated visually at pustulation stage and categorized as short, medium, or long. Panicle development was rated as “Good” if panicles were well-filled, compact, with plump, golden-glowing glumes and firm grains, and only a few sterile grains at the tip. Otherwise, it was rated as “Bad”. Floral organ abortion was recorded if spikelets were empty, with vestigial pistils or stamens, or if grain filling was terminated prematurely resulting in chaffy grains. Grain color was evaluated after threshing and classified as White, Yellow, or Brown. Data were recorded in Microsoft Excel and subjected to descriptive statistical analysis, including mean and standard deviation (SD) calculation. All experiments were performed with three biological replicates.

### 2.4 Development of a custom liquid-phase SNP panel for foxtail millet

#### 2.4.1 Selection of SNP markers and probe design

**Figure 2:**
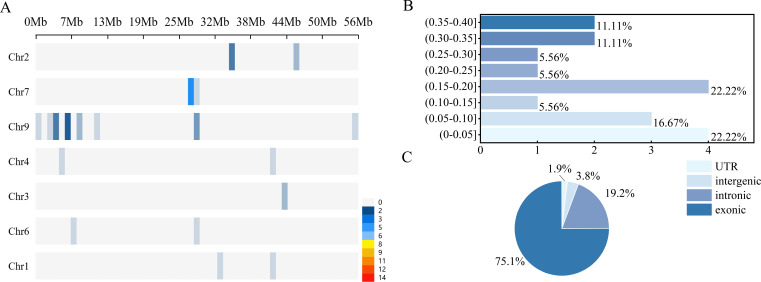
**(A)** Distribution of SNPs across genomic regions. The X-axis represents the physical location of loci on chromosomes, and Y-axis represents the chromosome numbers. **(B)** Distribution of SNPs by Minor Allele Frequency. The X-axis represents different MAF regions, and Y-axis represents the number. **(C)** Display the positions and distribution of SNPs on specific chromosomes.

The development of the custom SNP panel began with a comprehensive selection of high-quality SNP markers (**Figure 2**). This process integrated two primary strategies. First, we mined the literature for all available foxtail millet genome-wide association studies (GWAS) and reports on main-effect quantitative trait loci (QTLs) from databases including CNKI, PubMed, and Web of Science (**Supplementary Table 2**). QTL intervals that were consistently detected across multiple environments were prioritized. Genes within these conserved intervals were functionally annotated using the Phytozome database (Setaria italica v2.2), prioritizing candidate genes encoding transcription factors, protein kinases, and hormone metabolism-related proteins. SNPs were screened within the coding sequences (CDS), 5’ and 3’ UTRs, and promoter regions (2 kb upstream of the transcription start site). Priority was given to missense mutations and regulatory SNPs located in cis-acting elements.

Subsequently, we performed resequencing on a diverse panel of representative foxtail millet accessions to empirically validate and supplement the in silico selection. The resequencing data was aligned to the reference genome (Setaria italica v2.2) using BWA, and variants were called using GATK (Wang et al., 2019). From the pool of candidate SNPs identified through both literature mining and resequencing, redundant loci with low polymorphism (minor allele frequency, MAF < 0.05) or in high linkage disequilibrium (r² > 0.8) were excluded. Concurrently, we reviewed literature on millet genes from the past 20 years to compile a dataset of functionally characterized loci. The final panel integrated these sources, resulting in a refined set of high-confidence SNPs.

#### 2.4.2 Synthesis of the custom panel

The final list of selected SNP sequences and their flanking regions (used as capture probes) was submitted to Daicel Arbor Biosciences for the synthesis of the custom Liquid-Phase SNP Panel. The panel comprised biotinylated RNA oligonucleotides designed to specifically hybridize with the target genomic regions.

### 2.5 Genotyping of the foxtail millet population

#### 2.5.1 DNA extraction

After removing impurities and selecting seeds, approximately 10 seeds of each germplasm were planted in seedling trays and cultivated in a greenhouse. Each tray contained about 10 seeds. Samples were collected at the two-leaf stage and stored in 2 ml centrifuge tubes at -80 °C. Tender leaf (>0.3g) were collected, and plant DNA was extracted using a modified hexadecyl trimethyl ammonium bromide (CTAB) method. Genomic DNA concentration was measured using a Nanodrop 2000 spectrophotometer (Thermo Scientific, USA), with a threshold set at ≥20 ng/μL. DNA quality was further assessed by 1% agarose gel electrophoresis. Acceptable samples met the following criteria: OD_260_/_280_ > 1.8, OD_260_/_230_ > 1.5, and showed intact bands without significant degradation.

#### 2.5.2 Marker selection and primer design

A total of 59 functional markers previously associated with reproductive, Photosynthesis, leaf color, Flowering, Photoperiodicity, Biotic stress, Abiotic stress, quality, growth, plant type, panicle shape were selected from published studies. The original sequences of these markers were retrieved from public databases (NCBI GenBank) and relevant literature (see **Supplementary Table 1** for detailed information including original references, primer sequences, and expected amplicon sizes).

#### 2.5.3 PCR amplification and MassARRAY genotyping

Genomic DNA extracted in section 2.5.1 was used for SNP genotyping using the MassARRAY system (Agena Bioscience, USA) following the standard protocol. Primer design for PCR amplification and single-base extension was performed using Assay Design Suite v3.1 software (Agena Bioscience, USA) based on the flanking sequences of each SNP marker. PCR reactions were performed in 384-well plates with a total volume of 5 μL per well, containing 1× PCR buffer (with 1.5 mM MgCl_2_), 2.5 mM additional MgCl_2_, 0.5 mM each dNTP, 0.1 μM each forward and reverse primer, 0.5 U HotStar Taq DNA polymerase (Qiagen, Germany), and approximately 10 ng template DNA. A 38% reagent overage was included to account for pipetting losses. Thermal cycling conditions were: 94°C for 15 min; 45 cycles of 94°C for 20 s, 56°C for 30 s, and 72°C for 60 s; and 72°C for 3 min. Following PCR, unincorporated dNTPs were dephosphorylated using shrimp alkaline phosphatase (SAP) in a 2 μL reaction containing 1× SAP buffer and 0.3 U SAP, incubated at 37°C for 40 min followed by 85°C for 5 min. Single-base extension was performed using iPLEX Pro chemistry (Agena Bioscience, USA) in a 2 μL reaction containing 1× iPLEX buffer plus, 1× iPLEX termination mix, 0.6–1.3 μM extension primer mix, and 1× iPLEX enzyme, with thermal cycling of 94°C for 30 s; 40 cycles of 94°C for 5 s, 52°C for 5 s, and 80°C for 5 s (with 5 inner loops); and 72°C for 3 min. The final products (9 μL) were diluted three-fold, desalted with SpectroCLEAN resin, dispensed onto a 384-well SpectroCHIP array, and analyzed on a MassARRAY Analyzer 4 system with data collected using MassARRAY Typer 4.0 software (Kushwaha et al., 2023). Only markers showing clear, reproducible mass spectra and unambiguous genotype calls were used for subsequent analyses. All raw data and genotyping results are provided in **Supplementary Material**.

#### 2.5.4 Data analysis

Raw genotyping data generated by the MassARRAY system were processed using MassARRAY Typer 4.0 software to obtain SNP calls for all 192 accessions. Markers with call rate < 90% or monomorphic across all accessions were excluded from further analysis. Cluster analysis was performed using R software (R v.4.5.1). A hierarchical clustering tree was constructed using the ape package and visualized as a circular phylogram. The reliability of clustering was assessed by silhouette analysis using the cluster package.

### 2.6 Genetic stability and frequency

Minor allele frequency (MAF) is defined as the frequency at which the less common allele occurs in a given population. It is a fundamental metric in genetic studies for evaluating the population genetics of a variant and its suitability for association analyses. The frequency of minor alleles (MAF) was calculated for each locus. For common variants (MAF > 5%), univariate analysis is suitable. For rare variants (MAF < 5%), the statistical power of individual tests is low, and aggregation analysis (e.g., Burden Test, SKAT) is required to combine multiple rare variants within a single gene region for testing. A total of 19 variant sites were identified in the population. The presence of minor allele frequencies below 0.05 at certain loci reflects a narrow genetic background and skewed allele frequency distribution. The average site detection rate was 98.53% across all 192 accessions, indicating high genotyping success for the vast majority of samples. Notably, over half of the SNPs (52.3%) were low-frequency (MAF < 0.01). This enrichment of rare alleles likely reflects both artificial selection during domestication and the high homozygosity typical of self-pollination. This genetic architecture supports the use of univariate analysis for subsequent association studies.

## 3 Results

### 3.1 The locus statistics and genotyping effect of the chip

We successfully developed a high-density genotyping chip for targeted screening of functional loci governing key agronomic traits in millet. The chip was constructed based on a stringent selection process, focusing exclusively on loci with unequivocal evidence from either map-based cloning or well-characterized parental contributions in breeding populations. The final array integrates 59 optimally designed probes that collectively interrogate 30 functionally characterized gene loci (**Table 1**), with 1–3 SNPs per gene. These loci have been categorized into several major trait modules: (i) grain quality and composition, encompassing three loci for selenium accumulation; (ii) plant morphology and pigmentation, including 16 loci for leaf and sheath color, two for plant architecture, and one for spike morphology; (iii) yield-related traits, comprising two loci for grain size and four for reproductive development; (iv) stress adaptation, involving five loci for drought and salt tolerance; (v) developmental and physiological processes, covering four loci for growth and development, three for senescence regulation, five for photoperiod response, and seven for physiological performance; and (vi) one locus conferring fertility and two for disease resistance. Subsequently, we evaluated the custom SNP panel by genotyping 192 foxtail millet varieties with diverse genetic and geographical origins. After this strict quality control procedure, our platform demonstrated excellent genotyping efficiency. The site detection rate (the percentage of successfully typed sites) for each sample exceeded 75% across all varieties, and the overall genotyping success across rate across the population was high. Notably, the overall SNP call rate reached 98.53%, indicating extremely high data integrity and the reliability of the detection method.

**Table 1:** The loci and markers harbored in the breeder chip.

Trait type	Phenotype	Gene	Total loci
Yield	Grain Shape	SGD1	2
Reproductive	Development	MS1\FIE2\MADS34	4
	Senescence	YGL2	3
Photosynthesis/leaf color	Leaf Sheath color	PPLS1\WLS1	8
	Leaf color	PPLS1\CHL1\PCH1\LP1\WSL2	9
Flowering/Photoperiodicity	Flowering	PHYC\GI	5
Biotic stress	Downy Mildew	Seita.8G192100	2
	Development	AGO1\Seita.3G053400	2
Abiotic stress	Growth	Seita.6G095400	4
	Ni		1
	B	BOR1	2
	Drought tolerance	NP1	1
	Salt tolerance	STL2	2
Quality	Se	Seita.3G060000	3
	Grain color	PSY1	4
Growth	Growth	AUX1	3
	Polyploidy		1
Plant type/Panicle shape	Plant type	DPY1\BM1	2
	Panicle type	Si005660	1

### 3.2 Minor allele frequency spectrum

By calculating the frequency of the minor allele (Minor Allele Frequency, MAF) at each locus, we evaluated the genetic diversity of the loci in the 192 accessions (**Figure 2B**). We found that extremely rare variant sites in this group accounted for less than half of the total, and the minor allele frequency (MAF) of 22.2% of the single nucleotide polymorphisms (SNPs) was lower than 0.05. In contrast, the proportion of high-frequency alleles was relatively low. SNPs with MAF between 0.15–0.20, 0.20–0.25, and 0.35–0.40 each accounted for 5.6% of the total, while those between 0.25–0.30 and 0.30–0.35 each accounted for 11.1%. Together, these intermediate-frequency variants comprised less than 50% of all SNPs. This may be related to genetic selection during the domestication process of millet, as well as site variations and self-pollination.

### 3.3 Genomic distribution and functional annotation of SNP markers

**Figure 3:**
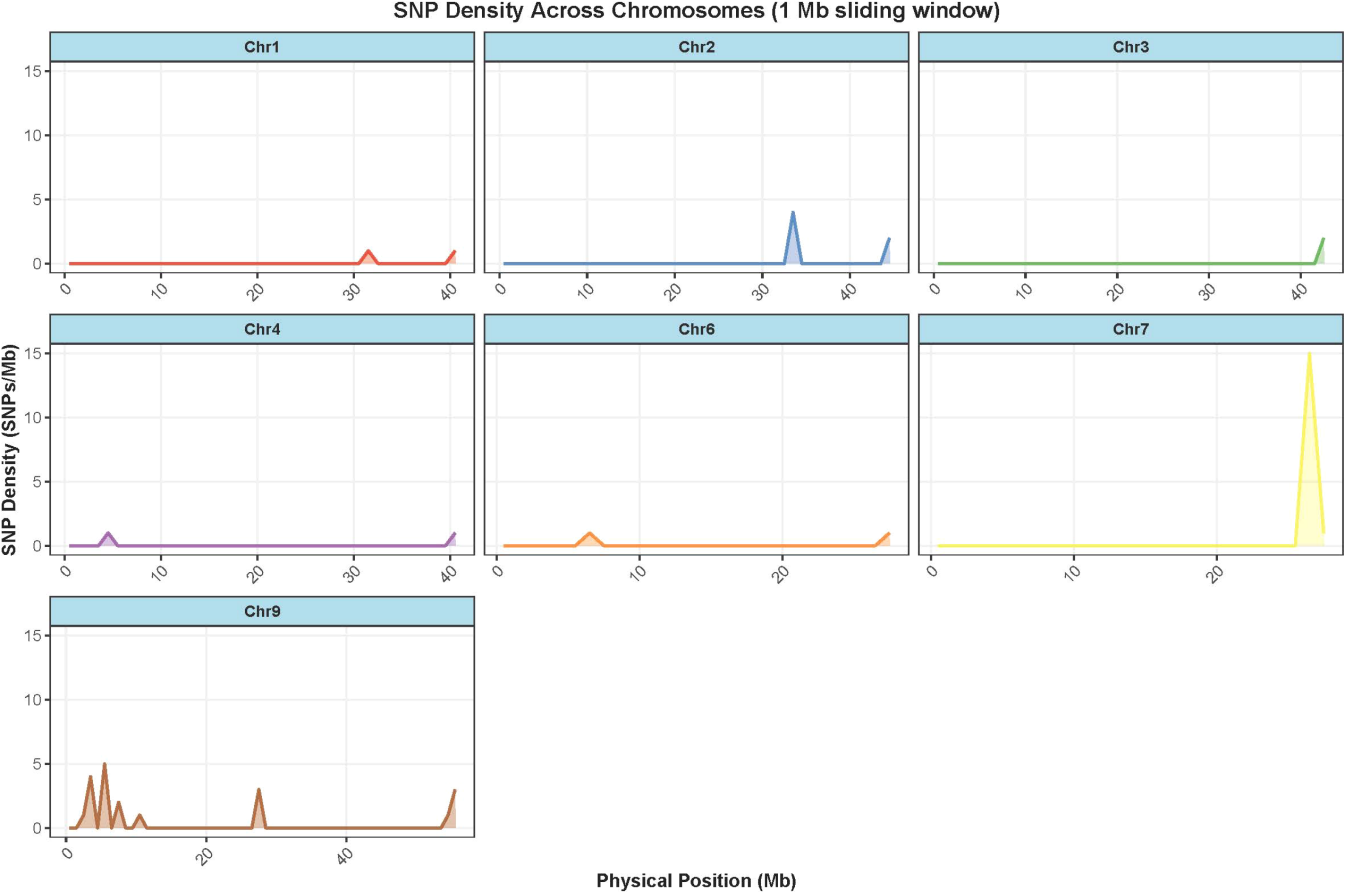
A displays the density of SNPs (number of SNPs per sliding window) across parts chromosomes.

A total of 59 SNP markers were successfully developed and characterized on the chip. To characterize the overall patterns of genetic variation, we examined the genome-wide distribution and density of SNPs. Their distribution across the foxtail millet (Setaria italica v2.2) genome was analyzed in 1-Mb windows. The SNPs were distributed on seven chromosomes (Chr1, 2, 3, 4, 6, 7, 9), with the number of SNPs per window ranging from 1 to 15 (**Figure 3**). Notably, chromosome 7 exhibited higher overall density, while Chr1, Chr3, Chr4 and Chr6 showed relatively low density. At a finer scale, we identified several prominent peaks of high SNP density, particularly in the pericentromeric regions of Chr7 and Chr9, suggesting these regions might experience higher rates of recombination or lower functional constraint.

Furthermore, chromosomes 1 to 9 exhibited a series of high-density peaks and low-density values, indicating that the distribution of genetic variations is uneven. For instance, a significant peak was observed at approximately position 28 Mb on chromosome 7, suggesting the presence of localized high polymorphism or reduced selective constraints in certain regions. Similar uneven distribution was also observed on other chromosomes. A bimodal distribution was also observed on chromosome 9, with the medium-density and low-density regions alternating. In contrast, the distribution patterns of Chr1, Chr2, Chr3, Chr4 and Chr6 were concentrated in only one or two regions and had a low density. The variation in genetic density observed in the genome was likely to reflect the interaction of multiple genomic processes, including differences in mutation accumulation, recombination rate heterogeneity, and variation in selective pressure across chromosomal regions. It is important to note that this analysis captures a regional cluster and does not reflect the genome-wide architecture of SNP distribution.

Functional annotation of the SNP locations revealed that the majority were located within genic regions (**Figure 2C**). Specifically, 39 SNPs (75.1%) were situated in exons, 10 (19.2%) in introns, and 1 (1.9%) in the 5’ UTR. The remaining 2 SNPs (3.8%) were mapped to intergenic regions. This distribution indicates a strong bias towards coding sequences, aligning with the strategy of targeting functionally relevant genes. The uneven genomic distribution of SNPs may be related to the enrichment of functional genes associated with agronomic traits, which was further verified in genotype-phenotype association analysis.

### 3.4 Phenotypic - genotype association analysis

**Figure 4:**
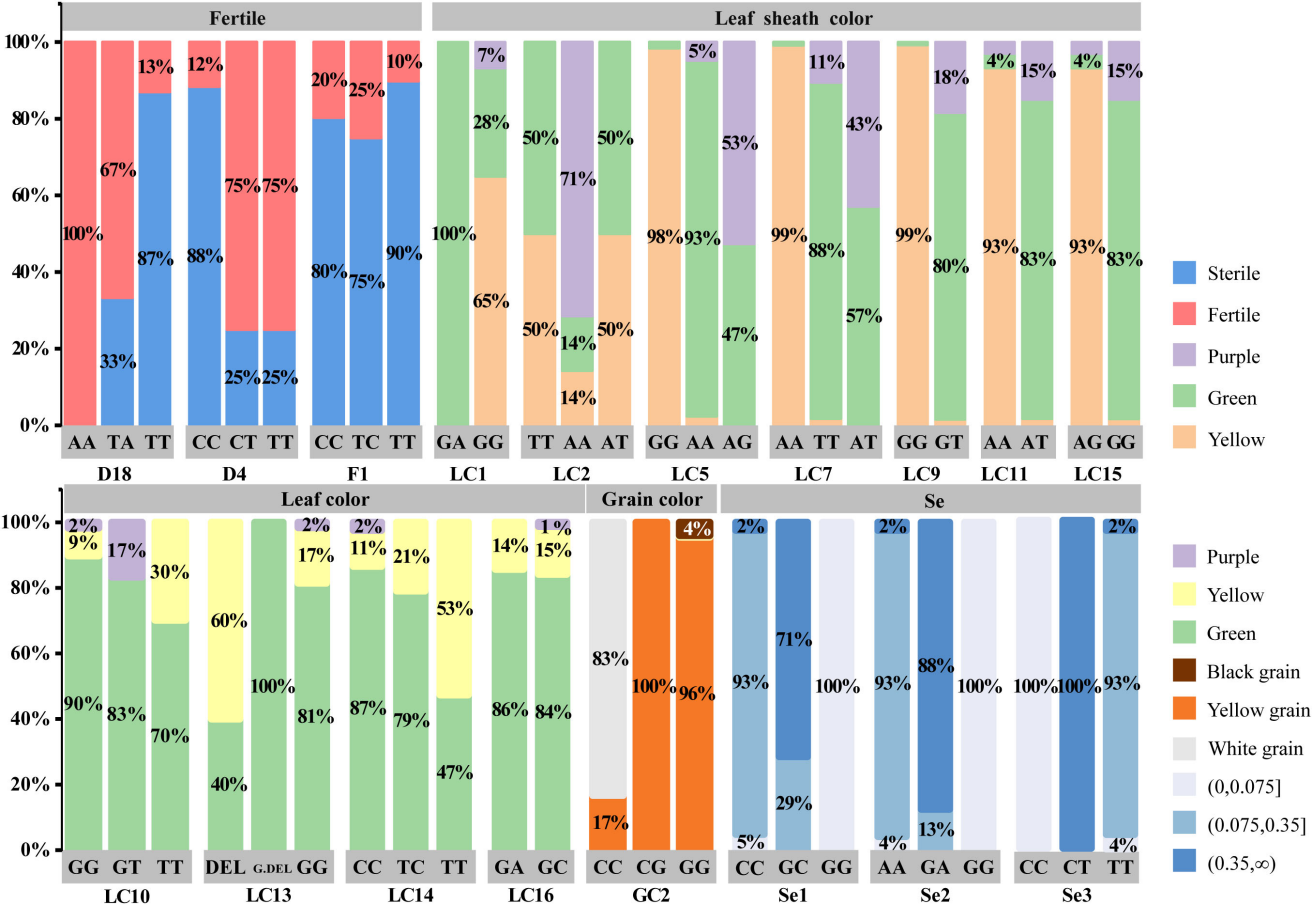
Genetic association of five Agronomic Traits. (1) Fertility, associated with loci including D18, D4, and F1 loci in the chip. (2) Leaf color during the growth period, associated with loci including LC10, LC13, LC14 and LC16 loci in the chip. (3) Leaf sheath color in seedlings, associated with loci including LC1, LC11, LC15 and LC9 loci in the chip. (4) Grain color, primarily associated with the GC2 locus. (5) The correlation of the Se1, Se2, Se3 loci in the chip for the selenium content in foxtail millet grains.

A total of 192 accessions, including wild accessions and major cultivated varieties widely used in foxtail millet breeding and cultivation, were selected as experimental materials. Through genetic association analysis, we assessed the correlations between five key agronomic traits—fertility, leaf color, leaf sheath color, grain color, and selenium accumulation—and specific molecular markers (**Figure 4**).

Genetic association analysis revealed three loci significantly associated with fertility. The D18 locus showed the strongest association, achieving an accuracy rate of over 87% in this population. The D2 locus had a concordance rate of approximately 75% with the phenotypic expression of fertility. Allele effects clarified the functional differences at these loci: varieties carrying the A allele could maintain normal fertility, while those carrying the T or C alleles exhibited infertility. The phenotypic expression of the T allele was more genetically distinct. D4 and D18 are tightly linked to the major genes controlling fertility recovery and sterility, respectively.

Analysis of the genotype-phenotype association of leaf sheath color revealed that the yellow leaf sheath phenotype had a strong correlation with the heterozygous GA genotype, while the green leaf sheath phenotype mainly corresponds to the homozygous GG or AA genotypes. The expression of purple leaf sheath color shows a more complex genetic pattern, mainly occurring in the heterozygous AG background, and frequently occurred on a green or yellow background – indicating incomplete dominance. Among the four significant association loci identified, LC9 showed the strongest influence ability. The variant was located in the exon region of the PPLS1 gene, which further supports the polygenic regulation of leaf sheath color in foxtail millet.

Genetic analysis of leaf color identified four loci (LC10, LC13, LC14, LC16), each with distinct phenotypic effects. There was a significant positive correlation between the green leaf color and the specific genotypes of these loci, while the yellow leaf phenotype is mainly associated with different genotype combinations. It was notable that the strength of the phenotypic association varied greatly among these loci, especially for LC10 and LC14. At these two loci, the GT and CC genotypes always result in purple leaf mutants, suggesting that they may play a key role in the regulation of leaf color changes or function as markers closely linked to the major effect genes. These findings collectively support the polygenic regulatory model for leaf color determination in foxtail millet, indicating the presence of conserved and actively expressed genetic components in the pigment biosynthesis pathway.

By analyzing the genetic characteristics of grain color, we found that this phenotype was closely related to the GC2 locus. The GG genotype was associated with the expression of white grains, while the CC genotype shows yellow grains. Most individuals with the CG heterozygous genotype exhibited yellow grains, but a brown-black grain was also observed, characterized by brown color with some yellowish hue. This suggests incomplete dominance or the influence of modifier factors. We noticed that varieties carrying two G alleles had a higher probability of developing black grain mutations. Preliminary analysis suggested that this may be caused by a specific base deletion—a phenomenon consistent with a potential gene dosage effect.

The correlation map analysis of selenium content in grains identified several significant genetic loci, namely Se1, Se2, and Se3. Genetic analysis revealed a clear allelic dose effect in selenium accumulation: at the Se1 locus, the homozygous GG genotype was associated with high selenium content (93% of the samples), while the heterozygous GA and homozygous AA genotypes corresponded to moderate levels (71%) and low levels (29%), respectively. Similar patterns were observed at the Se2 and Se3 loci, where the CC genotype promoted selenium accumulation, and the TT genotype showed extremely low content (2%). These results confirmed the polygenic structure of selenium accumulation, controlled by the synergistic action of multiple genetic loci. The major effects of Se1, Se2, and Se3 suggest that these loci play key regulatory roles in selenium metabolism, making it a preferred candidate gene for marker-assisted selection in nutritional breeding programs.

### 3.5 Evaluation of probe efficacy and genotyping accuracy in a SNP chip

**Figure 5:**
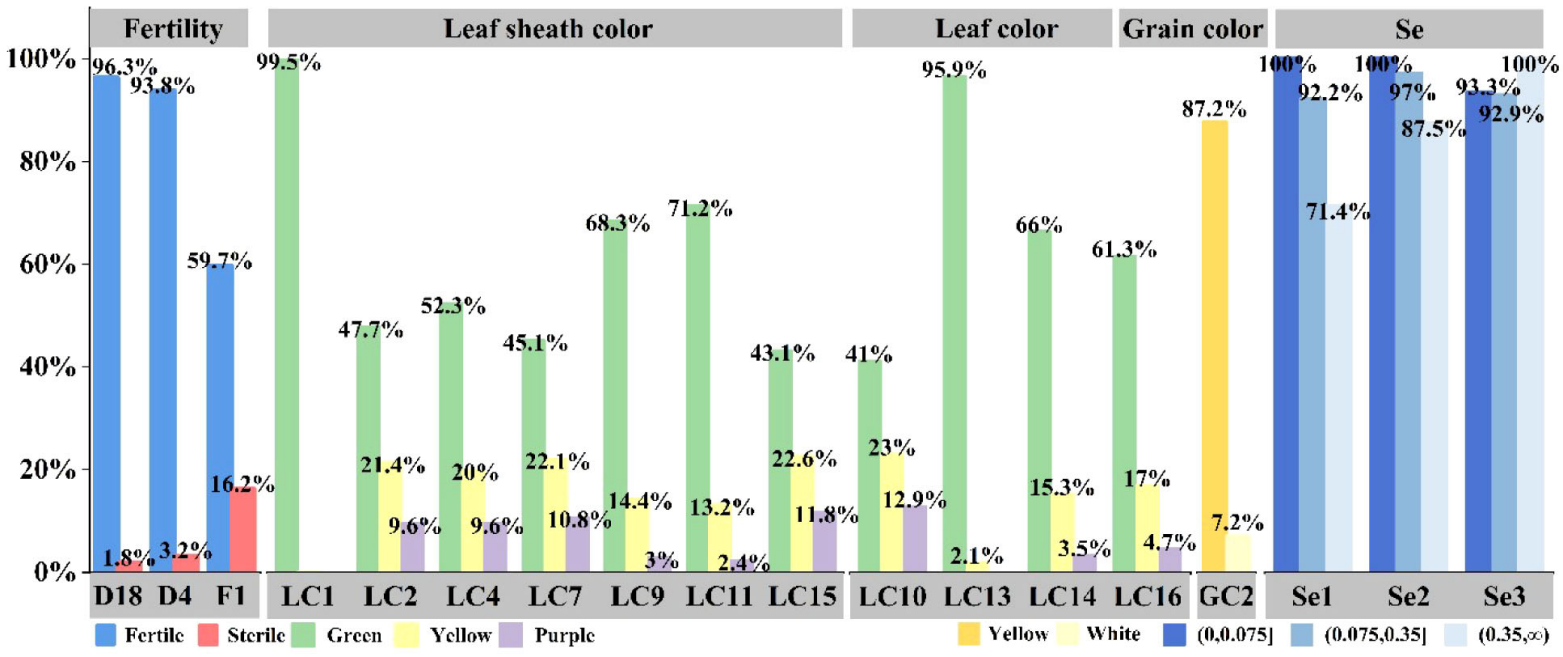
Detection efficiency of molecular maker for five agronomic traits. (1) Detection rates of the D18, D4, and F1 loci in the chip for the fertility of millet. (2) The detection rates of LC10, LC13, LC14 and LC16 loci in the chip for the leaf color of millet during the growth period. (3) The detection rate of LC1, LC11, LC15 and LC9 loci in the chip for the leaf sheath color of millet seedlings. (4) The detection rate of the GC2 loci in the chip for the color of millet grains. (5) The detection rate of Se1, Se2 and Se3 loci in the chip for the selenium content of grains.

Based on the genotyping results, the genotyping accuracy of the chip for four key agronomic traits in foxtail millet—seed setting rate, leaf color, leaf sheath color, and grain color—was evaluated (**Figure 5**). Through the analysis of breeding application, it was found that this chip had an accuracy of up to 96.3% in identifying fertile plants among individuals with normal fertility. However, the accuracy of the same genetic locus in identifying sterile plants was only 16.2%. This significant difference indicates that although this marker performed exceptionally well in tracking the dominant fertility alleles, it also suggests that sterility may involve a complex genetic structure beyond the scope of single-gene control—possibly including polygenic inheritance, epistatic interactions, or significant genotype-by-environment interactions.

The assessment of diagnostic loci for leaf color revealed significant differences in prediction accuracy among different phenotypic categories. The marker achieved an accuracy of up to 93.8% for the detection of green phenotypes, but the detection efficiency for yellow (59.7%) and purple (21.0%) variants gradually decreased. This stratified performance pattern indicates the existence of corresponding complexity in the genetic structure: the production of green pigments was mainly regulated by the target locus, while the formation of yellow and purple pigments may require complementary genetic components such as modifier genes, epistatic interactions, or modifiers of metabolic pathways, which greatly limits the resolution of single locus genotyping.

The molecular marker for leaf sheath color exhibited extremely high specificity, achieving near-perfect detection (99.5%) of the green sheath phenotype. The assessment of the identification of the leaf sheath color revealed that for non-green phenotypes, the utility of the loci was relatively reduced. The detection rate of yellow sheaths was below 22.6%, and that of purple sheaths was below 11.8%. This significant decline in utility indicates that there were different genetic structures for controlling the sheath color: the color of green sheaths seemed to be mainly controlled by the target locus, while the pigment synthesis of yellow and purple sheaths may involve multiple gene mechanisms, different biosynthetic pathways, or extensive epistatic interactions, which could not be captured by single-marker screening.

The analysis of the genetic characteristics of grain color revealed the effectiveness patterns of different markers. The diagnostic marker showed high specificity for detecting yellow grains (with a detection rate of 87.2%), confirming its effectiveness in identifying this major phenotype. In contrast, the detection rate for white grains was extremely low (7.2%), indicating that its association with the target locus was not complete. Notably, the black grain phenotype had a strong correlation with the specific base deletion at the GC2 locus, suggesting the existence of an alternative genetic mechanism that requires a specific molecular marker for accurate identification.

The genetic structure of selenium accumulation exhibited a context-dependent association pattern related to content. The chip showed a strong association with high-selenium varieties, achieving accurate genotype identification for Se3 (93.3%) and Se2 (92.9%). However, for the low-selenium Se1 material, the effectiveness of the marker significantly decreased (71.4%). This pattern indicates that under selenium-enriched conditions, the trait was primarily controlled by the target locus, whereas the low-selenium phenotype may involve modifier genes, epistatic interactions, or environmental factors that limit the efficiency of single-marker selection. Therefore, this chip is well-suited for marker-assisted selection in breeding programs targeting high selenium content.

### 3.6 Genetic cluster analysis

**Figure 6:**
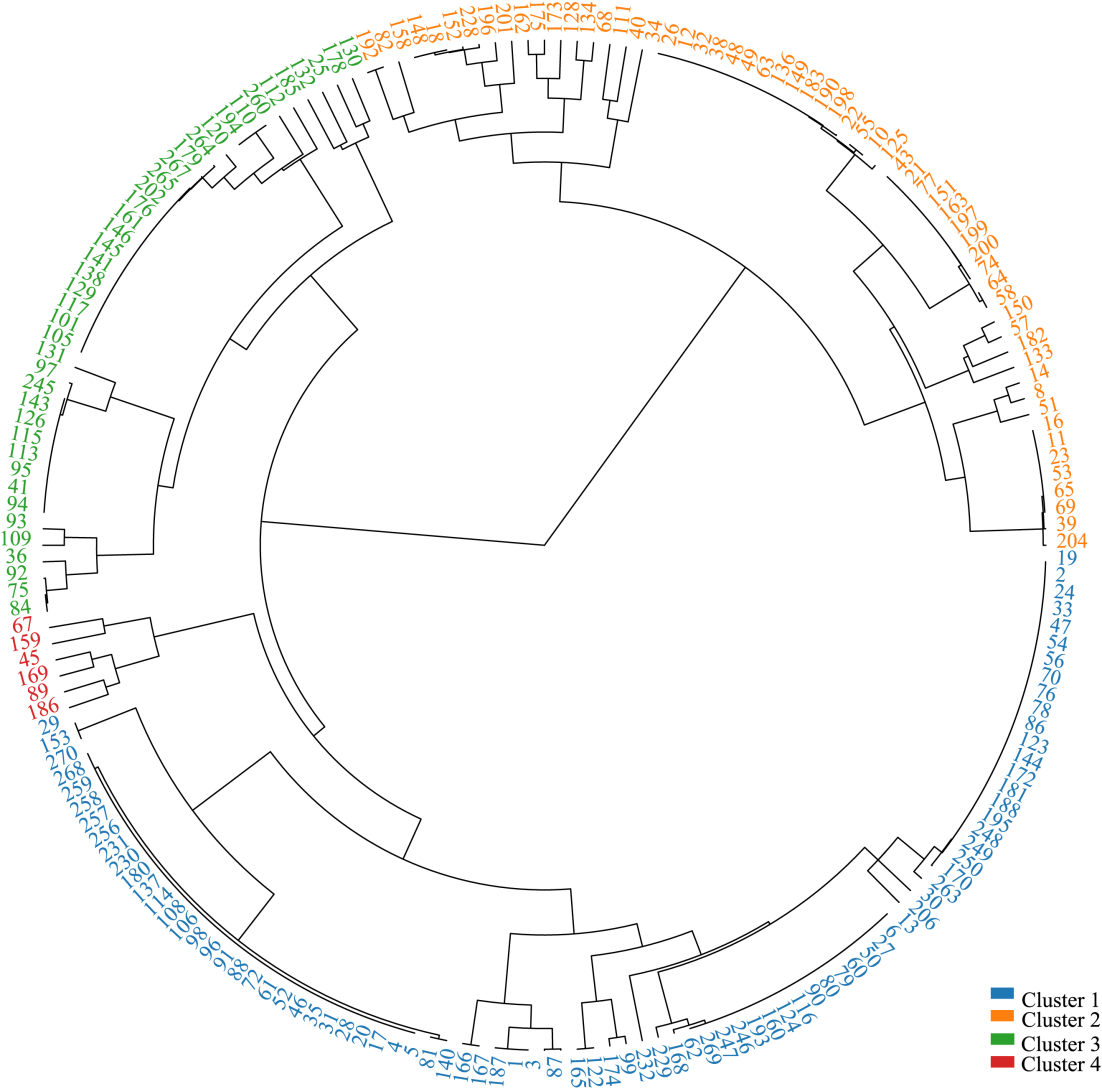
Phylogenetic evolutionary tree analysis of 192 foxtail millet varieties. (1) Cluster 1 in blue is a modern elite cultivar. (2) Cluster 2 in orange is a landrace. (3) Cluster 3 in green is an admixed group. (4) Cluster 4 in red is specific germplasm.

Based on the circular tree diagram of population clustering constructed using 52 conserved loci from 192 foxtail millet populations, a clear population stratification phenomenon was revealed (**Figure 6**). The clustering results confirmed the presence of genetically distinct subpopulations and provided an additional perspective for the genetic relationships within the foxtail millet germplasm resources.

The clustering analysis unambiguously classified the germplasm into four distinct genetic clusters. The phylogenetic tree revealed that Cluster 2 and Cluster 4 formed two deeply divergent and independent major branches, indicating that they represent landraces or germplasm with unique genetic backgrounds. In stark contrast, Cluster 1, a large clade comprising 94 accessions, exhibited very short internal branch lengths and a highly compact structure, strongly supporting its status as a group of modern elite cultivars with high genetic uniformity resulting from intensive selection. The phylogenetic position of Cluster 3 and the nesting of some of its accessions within Cluster 1 suggest it is an admixed group derived from gene flow between elite cultivars and landraces.

The phylogenetic tree confirmed from the perspective of evolutionary relationships that there was significant genetic differentiation in this millet population and precisely quantified the size of each group, providing practical guidance for the study of the evolutionary relationships of millet populations on chips.

## 4 Discussion

Foxtail millet stands as a nutrient-dense, drought-tolerant model cereal with great potential for sustainable agriculture, yet its genetic improvement has been consistently hampered by the lack of specialized genotyping tools. Prior to this study, foxtail millet breeding programs lacked effective tools for large-scale genetic analysis and marker-assisted selection. The liquid-phase functional SNP chip we developed filled this critical gap by integrating molecular genotyping with practical breeding applications. This customized platform targets 59 functionally validated SNPs and achieves 98.53% accuracy in genotyping 192 different varieties, while remaining cost-effective. Genetic analysis using this chip revealed distinct inheritance patterns. The traits of fertility and grain color exhibited simple monogenic genetic characteristics with high prediction accuracy, enabling efficient marker-assisted selection. I In contrast, leaf and leaf sheath color were controlled by polygenic inheritance, involving modifier genes and epistatic interactions, thus requiring a multi-marker selection strategy. Population structure analysis effectively distinguished modern cultivars, landraces, and admixed groups, providing valuable insights for parental selection and diversity management.

The flexible design of the GBTS platform allows for the continuous addition of newly identified functional markers, ensuring its long-term utility. By integrating technical reliability, functional relevance, and cost-effectiveness, this specialized genotyping tool can simultaneously screen for multiple agronomic, stress-resistance, and nutritional traits. This advancement is an important step towards cultivating millet varieties with adaptability and high yield and fully exploiting the potential of this ancient crop in sustainable agriculture.

## 5 Conclusion

The liquid-phase functional SNP chip developed filled this critical gap in foxtail millet breeding by integrating molecular genotyping with practical breeding applications. This customized platform targets 59 functionally validated SNPs and achieves an accuracy rate of 98.53% for genotyping 192 different varieties, while maintaining cost-effectiveness. Genetic analysis using this chip revealed various genetic patterns. The traits of fertility and grain color exhibited simple monogenic inheritance with high prediction accuracy, enabling efficient marker-assisted selection. In contrast, leaf and sheath color exhibited polygenic traits that involved modifier genes and epistatic interactions, thus requiring a multi-marker selection strategy. Population structure analysis effectively distinguished modern cultivated varieties, local varieties, and mixed populations, providing valuable insights for parent selection and diversity management. In summary, this study developed a foxtail millet GBTS liquid chip for a specific target population carrying functional SNPs related to agronomic traits. The constructed genotyping platform plays a significant role in germplasm evaluation, genetic diversity analysis, evolutionary analysis, and genomic selection, providing a powerful tool for precise breeding of foxtail millet. Beyond its utility in foxtail millet, this platform establishes a replicable technical paradigm—from functional marker discovery and customized chip design to multi-population validation and precision breeding—that can be readily adapted for other cereal crops. This advancement marks a critical step toward cultivating adaptable, high-yielding foxtail millet varieties and unlocking the full potential of this ancient crop in sustainable agriculture.

## Data Availability

The datasets presented in this study can be found in the article/**Supplementary Material**. The raw genotyping data generated using the MassARRAY system have been provided in **Supplementary Table S2**. All functional markers were derived from previously published sequences as cited in the text and **Supplementary Table S1**. Further inquiries can be directed to the corresponding author.

## References

[B1] Bennetzen J. L. Schmutz J. Wang H. Percifield R. Hawkins J. Pontaroli A. C. . (2012). Reference genome sequence of the model plant Setaria. Nat. Biotechnol. 30, 555–561. doi: 10.1038/nbt.2196, PMID: 22580951

[B2] Bettinger R. L. Barton L. Morgan C. (2010). The origins of food production in north China: A different kind of agricultural revolution. Evolutionary Anthropology: Issues News Rev. 19, 9–21. doi: 10.1002/evan.20236, PMID: 41848424

[B3] Burridge A. J. Wilkinson P. A. Winfield M. O. Barker G. L. A. Allen A. M. Coghill J. A. . (2018). Conversion of array-based single nucleotide polymorphic markers for use in targeted genotyping by sequencing in hexaploid wheat (Triticum aestivum). Plant Biotechnol. J. 16, 867–876. doi: 10.1111/pbi.12834, PMID: 28913866 PMC5866950

[B4] Campbell N. R. Harmon S. A. Narum S. R. (2015). Genotyping-in-Thousands by sequencing (GT-seq): A cost effective SNP genotyping method based on custom amplicon sequencing. Mol. Ecol. Resour. 15, 855–867. doi: 10.1111/1755-0998.12357, PMID: 25476721

[B5] Doust A. N. Kellogg E. A. Devos K. M. Bennetzen J. L. (2009). Foxtail millet: A sequence-driven grass model system. Plant Physiol. 149, 137–141. doi: 10.1104/pp.108.129627, PMID: 19126705 PMC2613750

[B6] Guo Z. Yang Q. Huang F. Zheng H. Sang Z. Xu Y. . (2021). Development of high-resolution multiple-SNP arrays for genetic analyses and molecular breeding through genotyping by target sequencing and liquid chip. Plant Commun. 2, 100230. doi: 10.1016/j.xplc.2021.100230, PMID: 34778746 PMC8577115

[B7] Johnson M. G. Pokorny L. Dodsworth S. Botigué L. R. Cowan R. S. Devault A. . (2019). A universal probe set for targeted sequencing of 353 nuclear genes from any flowering plant designed using k-medoids clustering. Systematic Biol. 68, 594–606. doi: 10.1093/sysbio/syy086, PMID: 30535394 PMC6568016

[B8] Kushwaha A. K. Ellur R. K. Maurya S. K. S G. K. Bashyal B. M. Bhowmick P. K. . (2023). Fine Mapping of qBK1.2, a Major QTL Governing Resistance to Bakanae Disease in Rice. Front. Plant Sci. 14, 1265176. doi: 10.3389/fpls.2023.1265176, PMID: 38023939 PMC10667430

[B9] Lata C. Gupta S. Prasad M. (2013). Foxtail millet: A model crop for genetic and genomic studies in bioenergy grasses. Crit. Rev. Biotechnol. 33, 328–343. doi: 10.3109/07388551.2012.716809, PMID: 22985089

[B10] Li X. Gao J. Song J. Guo K. Hou S. Wang X. . (2022). Multi-omics analyses of 398 foxtail millet accessions reveal genomic regions associated with domestication, metabolite traits, and anti-inflammatory effects. Mol. Plant 15, 1367–1383. doi: 10.1016/j.molp.2022.07.003, PMID: 35808829

[B11] Li Z. Jia Z. Li J. Kang D. Li M. Ma S. . (2024). Development of a 45K pepper GBTS liquid-phase gene chip and its application in genome-wide association studies. Front. Plant Sci. 15. doi: 10.3389/fpls.2024.1405190, PMID: 38984163 PMC11231373

[B12] Luo W. Tang Y. Li S. Zhang L. Liu Y. Zhang R. . (2023). The m6A reader SiYTH1 enhances drought tolerance by affecting the messenger RNA stability of genes related to stomatal closure and reactive oxygen species scavenging in Setaria italica. J. Integr. Plant Biol. 65, 2569–2586. doi: 10.1111/jipb.13575, PMID: 37861067

[B14] Samorodnitsky E. Jewell B. M. Hagopian R. Miya J. Wing M. R. Lyon E. . (2015). Evaluation of hybridization capture versus amplicon-based methods for whole-exome sequencing. Hum. Mutat. 36, 903–914. doi: 10.1002/humu.22825, PMID: 26110913 PMC4832303

[B15] Sharma N. Niranjan K. (2018). Foxtail millet: Properties, processing, health benefits, and uses. Food Rev. Int. 34, 329–363. doi: 10.1080/87559129.2017.1290103, PMID: 41799851

[B17] Unterseer S. Bauer E. Haberer G. Seidel M. Knaak C. Ouzunova M. . (2014). A powerful tool for genome analysis in maize: Development and evaluation of the high density 600 k SNP genotyping array. BMC Genomics 15, 823. doi: 10.1186/1471-2164-15-823, PMID: 25266061 PMC4192734

[B18] Verlouw J. A. M. Clemens E. de Vries J. H. Zolk O. Verkerk A. J. M. H. Am Zehnhoff-Dinnesen A. . (2021). A comparison of genotyping arrays. Eur. J. Hum. Genetics: EJHG 29, 1611–1624. doi: 10.1038/s41431-021-00917-7, PMID: 34140649 PMC8560858

[B19] Wang X. Deng Z. Hu Y. Rehman F. An Z. Wu T. . (2025). Development of the rubber tree 40K breeding chip with applications in genetic study and breeding prediction. Ind. Crops Products 226, 120640. doi: 10.1016/j.indcrop.2025.120640, PMID: 41853590

[B20] Wang Z. Wang J. Peng J. Du X. Jiang M. Li Y. . (2019). QTL mapping for 11 agronomic traits based on a genome-wide Bin-map in a large F2 population of foxtail millet (Setaria italica (L.) P. Beauv). Mol. Breed. 39, 18. doi: 10.1007/s11032-019-0930-6, PMID: 41853694

[B21] Xiang M. Liu S. Wang X. Zhang M. Yan W. Wu J. . (2023). Development of breeder chip for gene detection and molecular-assisted selection by target sequencing in wheat. Mol. Breeding: New Strategies Plant Improvement 43, 13. doi: 10.1007/s11032-023-01359-3, PMID: 37313130 PMC10248658

[B22] Xing G. Zhang L. Feng W. Xie S. Jia Y. Yuan Y. . (2016). Analysis ofWRKY transcription gene family in foxtail millet. J. Shanxi Agric. Univ. (Natural Sci. Edition) 36:837–845.

[B23] Yang Q. Zhang J. Shi X. Chen L. Qin J. Zhang M. . (2023). Development of SNP marker panels for genotyping by target sequencing (GBTS) and its application in soybean. Mol. Breeding: New Strategies Plant Improvement 43, 26. doi: 10.1007/s11032-023-01372-6, PMID: 37313526 PMC10248699

[B24] Yang X. Wan Z. Perry L. Lu H. Wang Q. Zhao C. . (2012). Early millet use in northern China. Proc. Natl. Acad. Sci. 109, 3726–3730. doi: 10.1073/pnas.1115430109, PMID: 22355109 PMC3309722

[B25] Zhang G. Liu X. Quan Z. Cheng S. Xu X. Pan S. . (2012). Genome sequence of foxtail millet (Setaria italica) provides insights into grass evolution and biofuel potential. Nat. Biotechnol. 30, 549–554. doi: 10.1038/nbt.2195, PMID: 22580950

