# Supplementary material for: Development of liquid chip for functional genes in foxtail millet (Setaria italica) and association analysis of related traits
Source: Front Plant Sci. 2026 Mar 24;17:1784690. doi: 10.3389/fpls.2026.1784690 (PMC13053507; doi:10.3389/fpls.2026.1784690)
Supplement: Supplementary file 1 [file Image1.pdf]

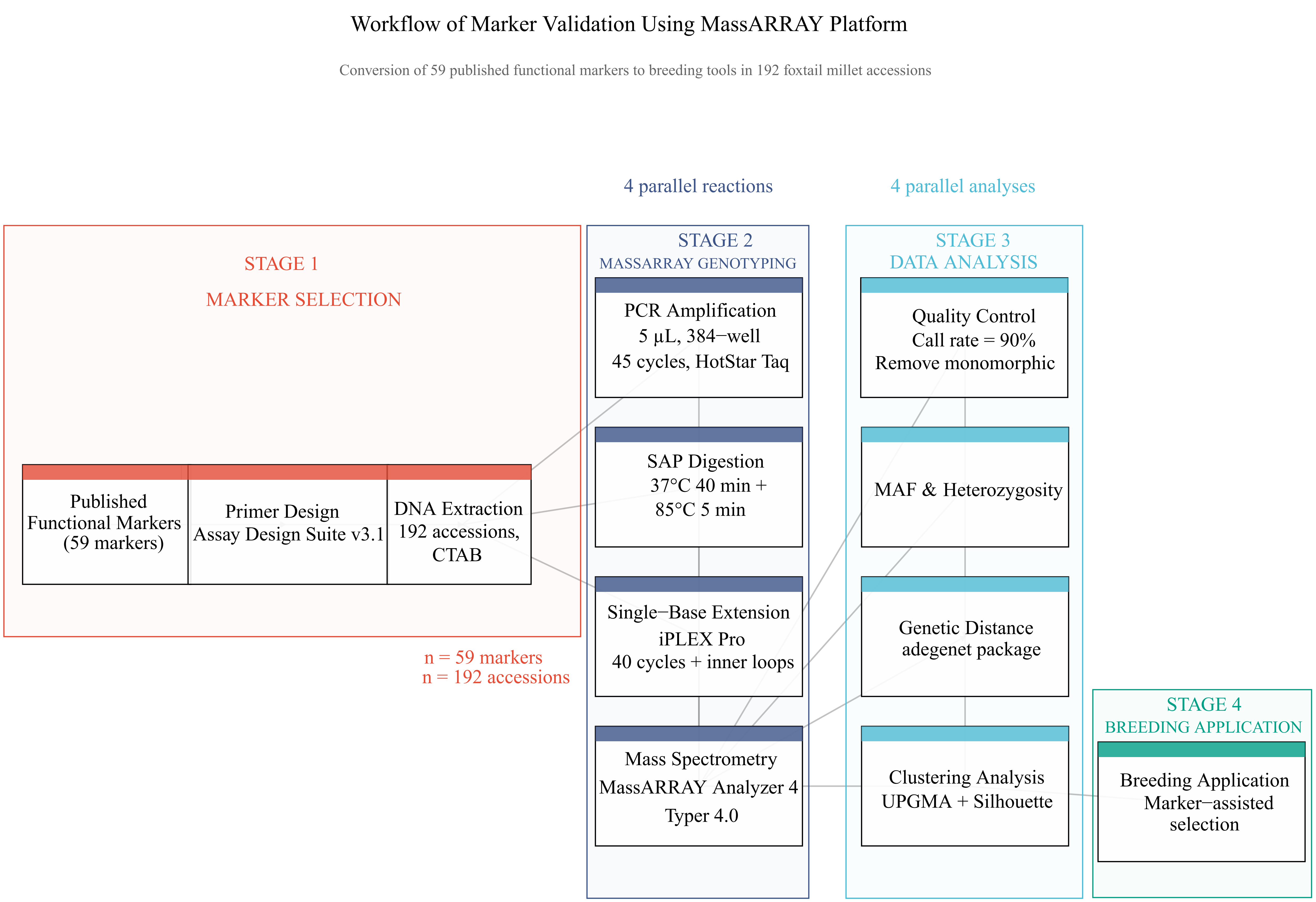
Workflow of Marker Validation Using MassARRAY Platform
Conversion of 59 published functional markers to breeding tools in 192 foxtail millet accessions
4 parallel reactions
4 parallel analyses
STAGE 1
MARKER SELECTION
Published Functional Markers (59 markers)
Primer Design Assay Design Suite v3.1
DNA Extraction 192 accessions, CTAB
n = 59 markers
n = 192 accessions
STAGE 2
MASSARRAY GENOTYPING
PCR Amplification 5 µL, 384–well 45 cycles, HotStar Taq
SAP Digestion 37°C 40 min + 85°C 5 min
Single–Base Extension iPLEX Pro 40 cycles + inner loops
Mass Spectrometry MassARRAY Analyzer 4 Typer 4.0
STAGE 3
DATA ANALYSIS
Quality Control Call rate = 90% Remove monomorphic
MAF & Heterozygosity
Genetic Distance adegenet package
Clustering Analysis UPGMA + Silhouette
STAGE 4
BREEDING APPLICATION
Breeding Application Marker–assisted selection
